# How criminal science publishing gangs damage the genesis of knowledge and technology—a call to action to restore trust

**DOI:** 10.1007/s00210-021-02158-3

**Published:** 2021-09-23

**Authors:** Bernhard A. Sabel, Roland Seifert

**Affiliations:** 1 Institute of Medical Psychology, Otto-V.-Guericke University of Magdeburg Medical School, Leipzigerstr. 44, 39120 Magdeburg, Germany; 2 Institute of Pharmacology, Hannover Medical School, Carl-Neuberg-Str. 1, 30625 Hannover, Germany

## What is the power of criminal science publishing gangs ?

Criminal science publishing gangs—formerly known as “paper mills”—are fraudulent businesses producing fake scientific papers with false content. We decided to use the former term because the latter one was inspired by a fantasy novel (Byrne and Christopher 2020). But we are not dealing with a fantasy novel. We are dealing with the reality of true crime.

Fraudulent science papers are produced by scientists who sell their services of writing ghost papers for customers who are presumably in urgent need to demonstrate their scientific output. Their aim is to generate—or polish—their “scientific standing” to personally gain job promotions and increase their “credibility.” Criminal science publishing gang products are sold in many scientific disciplines and challenge the very foundation of trust and reliability of scientific conduct. Such publications are often hard to identify because they are written by trained scientists. Accordingly, their apparent quality is often high enough to end up being published in scientific peer-reviewed journals (Seifert 2021) and thus become part of the permanent scientific record. The mechanisms of operation of criminal science publishing gangs are now well understood and have been recently reviewed (Christopher 2021; Seifert 2021). Yet, the problem is growing at a concerning rate. To help solve the problem, two Editors-in-Chief of scientific journals (Bernhard A. Sabel, *Restorative Neurology and Neuroscience*; and Roland Seifert, *Naunyn–Schmiedeberg’s Archives of Pharmacology*) have joined up to write this Editorial and voice an urgent call to action.

## Of truth and trust

Journal editors, scientific peer reviewers, and journal publishers work under the assumption of trust that authors report data correctly and interpret them fairly in an unbiased manner in the context of the current state-of-the-art. Trust is the fundamental value system of the entire scientific community which aims to promote our knowledge on the basis of honest experiments, observations, and analyses. If there is no trust, there is no truth. Unveiling scientific misconduct is therefore an essential element of science. If this system of trust is violated, it will create confusion and waste human and financial resources, carrying great risks in health, technology, environment, and humanity at large. As in personal and business relationships, trust is slowly gained, but it can be quickly lost.

Scientific misconduct, producing false data or faking publications, has always been there in the history of science, with many cases of fraud where scientists failed to follow the rules of “good scientific practice,” yet succeeded to publish papers of fake and fiction in (often high profile) journals (van der Heyden 2021). But recently, this problem has got out of control and is going “global”: in the past couple of years, an entire industry has emerged to intentionally produce and sell fake papers simply for mundane career reasons, deceiving the scientific community in all countries around the globe with a dramatic impact on science.

Although we do not know the precise number of such criminal science publishing gangs and which countries are affected by them, China has been the most prominent location of such practices. Its science administration has started to recognize the impact of this problem and addressing it by circulating a “Blue Book” with the aim to reduce or stop such activities. http://www.naturechina.com/pdf?file=/public/upload/pdf/2021/04/02/60667baa40ebc.pdf. This was co-published by Springer Nature China and the Institute of Scientific and Technical Information of China (ISTIC), the national agency providing intelligence to the Ministry of Science and Technology.

Yet, especially in the medical field, criminal science publishing gangs are churning out more and more fake papers (hence the earlier use of the term “paper mill”). They are being paid for their services by scientists and clinicians who struggle with increasing professional pressures. Their working days are filled with clinical services of treating patients, yet they are also expected to be scientifically “productive” to fulfill their employer’s often unrealistic goals (Else and van Noorden 2021). It is this expectation pressure (“push factor”) together with the seduction by criminal science publishing gangs (“pull factor”) which provides the breeding ground for deception. While the individual scientist feels he or she benefits (receiving promotion or increasing credibility and reputation), it is the institution, the respective country, and global scientific community who lose (trust).

Criminal science publishing gangs have become a gigantic fraud operations and their damage is just beginning to unfold. But it is not too late, we hope. One journal, *Naunyn–Schmiedeberg’s Archives of Pharmacology*, the oldest existing pharmacological journal of the world, has become a prime target of science criminal gang publications (Seifert 2021). The journal has a long tradition of publishing high-quality papers in the field of phytopharmacology, a topic popular in Chinese medical sciences because of the country’s long history of traditional Chinese medicine (TCM). Thus, *Naunyn–Schmiedeberg’s Archives of Pharmacology* is a logical target for the criminal science publishing gangs because of the combination of job pressure of medical scientists and the Chinese medical science focus on TCM and natural drugs.

Over the past years, *Naunyn–Schmiedeberg’s Archives of Pharmacology* noticed a very substantial increase in submissions from China. Many of them passed stringent peer review and were subsequently published. However, in early 2020, the Editor-in-Chief became aware of irregularities in publications based on duplicate images. This triggered the investigation of all suspected cases and the editor requested original source data of tables and figures. Papers without original data were no longer published. This has resulted in 14 retractions so far and the (often “last-minute”) pre-publication retraction of 30 papers (authors failed to provide original data) after they had been already accepted. Originally, it was estimated that about 5% of all submissions to the journal were fake (Seifert 2021). While this number is substantial, nonetheless, it is still just a small fraction of all submissions.

But the situation has recently turned out to be much worse: a careful analysis of the metrics of *Naunyn–Schmiedeberg’s Archives of Pharmacology* for 2020 revealed that 320 additional papers, virtually all from China, were withdrawn because they failed to reply to the editor’s request to provide original data. This means that 30% of all submissions to this journal in 2020 were likely fake. None of the withdrawn papers was co-authored by an international research consortium, indicating that the lack of external input and communication might have fostered fraud. Unfortunately, at least some fake papers withdrawn in *Naunyn–Schmiedeberg’s Archives of Pharmacology* ended up being published in other journals, with a slightly modified title but completely different authors (Seifert 2021). Other journals have suffered similar criminal science publishing gang attacks (Seifert 2021; Christopher 2021) such as the journal *Molecular Brain* which estimates a similar figure of 25% fake submissions (Miyakawa 2020). These very high numbers are extremely disturbing and underline the severity of the problem. In addition, the limited data base reveals that fake science does not only affect phytopharmacology but also other scientific fields, especially medical science, including cancer research and neuroscience (Miyakawa 2020; Christopher 2021; Heck et al. 2021).

## The scope of the problem

The magnitude of fraud is shocking and has the potential to not only ruin the reputation of the “black sheep” scientists, but it has the potential to ruin the efforts of all “white sheep” scientists who struggle daily with their important and highly credible work. However, it is still unclear if and to what extent different journals, scientific disciplines, or countries are affected by criminal science publishing gangs. We are afraid that many journals have not yet fully recognized the huge scope of the problem or have not yet issued official statements on whether they too are victims of such fraud.

Given the limited database today, it is exceedingly difficult to assess how badly the scientific literature is contaminated. Given the strong increase of false submissions from China to scientific journals over the past decade, it was proposed that most such gangs are located in China (Else and Van Noorden 2021). Yet, to learn which institutions are the perpetrators mandates a detailed analysis by their respective country. We may just see the tip of the “icebergs of fake” and are probably not at the bottom of the pit yet. We hope that relevant authorities initiate measures to eradicate this industry-style dismantling of trust in science.

## The therapy for fraud and its prevention

Prevention of fraud is in the best interest of all stakeholders. To regain the trust in science is not only a problem for China but also other countries or—in a worst-case scenario—the scientific community at large. Whatever the scope, there are solutions to the problem—and rectifying the situation immediately is needed to prevent fake science to spread around like an uncontrolled virus epidemic.

Solutions involve different measures on the level of scientists and their employers, journal editors and publishers, and the local and national government bodies (Table 1). At the level of journal editors, *Naunyn–Schmiedeberg’s Archives of Pharmacology* serves as a role model how stringent submission requirements of providing original data at submission can lead to a rapid and substantial decrease in false submissions. The preliminary analysis of this journal in 2021 indicates that the total number of submissions will remain at the level of 2020 if one substracts the approximately 350 fake submissions from China in that year. These few bibliometric numbers suggest that its Editor-in-Chief has identified and substantially solved the fake paper problem.

**Table 1:** Prevention and therapy of science fraud

**1. Quality control by editors and journals** Pre-publication surveillance • Provide a declaration that the data and analysis were done according to good scientific practice and that the data and their analysis are real • Rejection of private email addresses, professional emails required for all authors • Authors have to provide original data and images already with the initial submission • First, senior, and corresponding authors provide confirmation of their employment and contact information by their institutional leadership (e.g., dean) • Listing verifiable grant support sources • Corresponding author should be required to apply for and list their ORCID registration Post-publication peer review • Double check if email is institutional (not private) • In case of doubt, request post-publication proof of data and images • Check for duplicate publications in other journals **2. Institutional quality control** • Teaching and controlling guidelines for good scientific practice • Remove incentives for overly ambitious scientists or pressure of institutions (such as hospitals) • Avoid unrealistic publication pressures (for example, by clinics or institutions) • Eliminate the sole use of impact factors (IF) and the sheer number of papers as a “quality” measure of scientific productivity • Focus more on the long-term impact of research projects • Encourage team-based international collaborations (checks-and-balances) **3. Quality control by publishers and governmental bodies** • Central registry of ORCIDs (international science IDs) and individual publication record using digital object identifier (DOI) • Outlaw those who operate criminal science publishing gangs; legal prosecution for damages • Sanctions for scientific misconduct • Increase funding for young scientist and give them more independence • Create a black-list registry of scientific misconduct (“bad banks”), where authors, collaborating journals, and institutions are listed • Streamline counter-measures among all stakeholders and journals worldwide	

Besides requesting original data upon submission, journal editors could also adopt other countermeasures. They include a range of different measures such as surveillance, plausibility checks, ORCID-requirements, confirmations from institutions (e.g., Dean) that the scientist is actually employed by the institution, submission of publication lists of authors, acceptance of first authors’ institutional email addresses only (rejection of private email addresses), full email disclosure of all co-authors, and declarations of strict adherence to responsible scientific conduct, including submission of original data such as Western blots and fluorescence images (Table 1).

When integrity is in doubt, already published papers must be withdrawn if authors cannot—or are unwilling to—provide original data or other information needed for plausibility checks. These (unfortunately very time-consuming and annoying) defense measures are already applied successfully in *Naunyn–Schmiedeberg’s Archives of Pharmacology* (Seifert 2021). The feedback from the “white sheep” scientific community was very encouraging and shows that it values the stringent journal response to restore trust.

Some journals or publishers may be reluctant to implement rigorous decontamination operations in their published and unpublished records because of lack of personnel and/or financial resources. Or they fear to lose reputation and receive fewer submissions which might impact publishers’ revenue. However, based on the recent experiences with *Naunyn–Schmiedeberg’s Archives of Pharmacology*, we strongly encourage all journal editors and their publishers to rigorously analyze their published papers and unpublished submissions to assess the true dimension of the fake paper crisis. Without this very painful and time-consuming process, the publishers and the entire scientific community will waste financial resources and lose time in the long run. Namely, by planning new scientific experiments on the basis of fake data not only jeopardizes the careers of junior scientists, but it also reduces a country’s productivity in science and technology. The experience with *Naunyn–Schmiedeberg’s Archives of Pharmacology* shows that the faster and more complete the decontamination process is performed, the earlier this nightmare can be stopped. This, in turn, helps to protect a journal’s solid reputation and avoids that the journal ends up with hundreds of retractions. And it will shield publishers and science administrations from embarrassment.

Journal decontamination and adjustments in science and institutional policies are not something to be ashamed of and the changes should be quick and effective. Otherwise, the risk for a loss of reputation is long lasting if criminal science publishing gangs or administrations are being caught for bending or hiding the truth. Scientists, publishers, and administrators alike are therefore well advised to openly face the problem and fixing it. It is a professional move in the right direction if journals, publishers, and science administration bodies have the courage to openly communicate that they are the victims being targeted by criminal science publishing gangs. Then, they are free to act swiftly to stop all persons or agencies operating such criminal science publishing gangs. These gangs are “professionals” and they know very well how to play the game and fool honest scientists industrial-style.

## Impact on science and society

Journals and publishers should not hesitate to clean up their publication records. History has taught us a most painful lesson in Germany: *Naunyn–Schmiedeberg’s Archives of Pharmacology*, before 1933, had been one of the most prestigious pharmacological journals with many Nobel prize winners publishing their top papers in this journal, but the journal only barely survived the grueling attack by Nazi regime’s science authority on pharmacology in Germany (Starke 1998). It took decades to rebuild the journal’s reputation, and the process is still ongoing. Likewise, had the massive fake paper attack on the journal in 2019–2020 not been recognized and diligently counteracted immediately, the world’s oldest pharmacological journal would have probably been pushed over the cliff and died before its 150th anniversary in 2023. But criminal science publishing gangs do not care about history. Also, with the reunification of Germany in 1990, the scientific community of the former German Democratic Republic (GDR) which was heavily contaminated by non-scientific, political influences, needed to be completely restructured to become internationally competitive again (Sabel 1993). So, both authors of this Editorial are sensitized by their experiences with the value of ethics of good scientific conduct and the (re-) structuring of science systems and their operation.

Honest science by “white sheep” scientists is also in the best interest of employers, publishers, and governments. In contrast, fake “black sheep” scientists and their publications can lead to disaster, not only because they contaminate the knowledge base of humanity, but their behavior can impact many areas of everyday life such as health care and technology development. Especially in medicine, wrong information can cost lives. But in the end, only diligent and honest science will overcome the greatest challenges and prevail. Fellow journal editors and publishers should therefore not hesitate to investigate their own journals and step forward in the fight against science publishing gangs. We call upon all journals, employers, publishers, and government bodies to stamp out this pandemic of fake science.

The Chinese Government has already taken first steps to address the problem with the “Blue Book” (see above). Time will show if this is enough to turn the situation around, but more measures are probably needed. If these or similar interventions are not effective, criminal science publishing gangs can quickly ruin the reputation of academic institutions, administrative organizations, publishers, or countries, hindering global progress of science and technology more so than common criminals. Scientists, journal editors, publishers, and administrators should not be afraid to lose face but rather face the ruthless reality. The time to show courage and take action to regain the trust we all deserve is now!

What do science and “true love” have in common? Both are infatuated by passion, but if trust is lost, it is almost impossible to go back.
